# (Acetato-κ^2^ *O*,*O*′)[2′-(di-*tert*-butyl­phosphanyl)-1,1′-biphenyl-κ^2^ *P*,*C* ^2^]palladium(II)

**DOI:** 10.1107/S1600536812040068

**Published:** 2012-09-26

**Authors:** Charmaine Arderne, Cedric W. Holzapfel

**Affiliations:** a Department of Chemistry, University of Johannesburg, PO Box 524, Auckland Park, Johannesburg 2006, South Africa

## Abstract

The structure of the title compound, [Pd(C_2_H_3_O_2_)(C_20_H_26_P)], shows a distorted square-planar geometry for the Pd^II^ atom, with significant deviations being evident owing to the asymmetric coordination mode of the acetate ligand. A weak intra­molecular C—H⋯O inter­action is noted. The crystal studied was a racemic twin.

## Related literature

For related structures and catalytic literature on palladium complexes, see: Ormondi *et al.* (2011 ▶); van Blerk & Holzapfel (2009 ▶); Zim & Buchwald (2005 ▶); Williams *et al.* (2008 ▶).
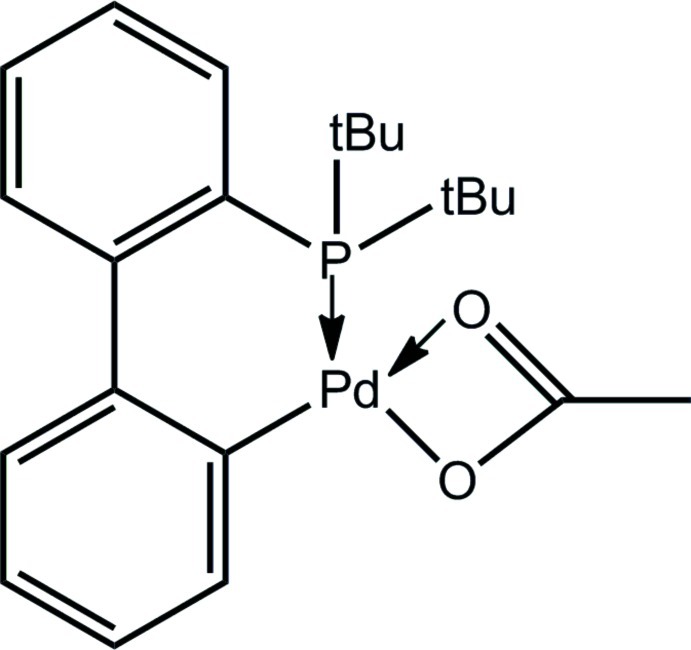


## Experimental

### 

#### Crystal data

[Pd(C_2_H_3_O_2_)(C_20_H_26_P)]
*M* *_r_* = 462.82
Orthorhombic, 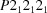

*a* = 9.800 (3) Å
*b* = 14.2392 (4) Å
*c* = 14.7772 (5) Å
*V* = 2062.1 (6) Å^3^
*Z* = 4
Mo *K*α radiation
μ = 0.99 mm^−1^
*T* = 100 K
0.25 × 0.19 × 0.14 mm

#### Data collection

Bruker APEXII CCD diffractometer
Absorption correction: multi-scan (*AXSCALE*; Bruker, 2010 ▶) *T*_min_ = 0.790, *T*_max_ = 0.874
24048 measured reflections
5179 independent reflections
5077 reflections with *I* > 2σ(*I*)
*R*_int_ = 0.018

#### Refinement

*R*[*F* ^2^ > 2σ(*F* ^2^)] = 0.014
*wR*(*F* ^2^) = 0.034
*S* = 1.06
5179 reflections
243 parameters
H-atom parameters constrained
Δρ_max_ = 0.27 e Å^−3^
Δρ_min_ = −0.19 e Å^−3^
Absolute structure: not determined

### 

Data collection: *APEX2* (Bruker, 2010 ▶); cell refinement: *SAINT* (Bruker, 2010 ▶); data reduction: *SAINT*; program(s) used to solve structure: *SHELXS97* (Sheldrick, 2008 ▶); program(s) used to refine structure: *SHELXL97* (Sheldrick, 2008 ▶); molecular graphics: OLEX2 (Dolomanov *et al.*, 2009 ▶); software used to prepare material for publication: *PLATON* (Spek, 2009 ▶) and *publCIF* (Westrip, 2010 ▶).

## Supplementary Material

Crystal structure: contains datablock(s) I, global. DOI: 10.1107/S1600536812040068/tk5150sup1.cif

Supplementary material file. DOI: 10.1107/S1600536812040068/tk5150Isup2.mol

Structure factors: contains datablock(s) I. DOI: 10.1107/S1600536812040068/tk5150Isup3.hkl

Additional supplementary materials:  crystallographic information; 3D view; checkCIF report

## Figures and Tables

**Table:** 

Pd1—P1	2.2285 (7)
Pd1—O1	2.2109 (12)
Pd1—O2	2.1657 (11)
Pd1—C18	1.9686 (15)

**Table:** 

P1—Pd1—O1	113.61 (3)
P1—Pd1—O2	173.58 (3)
P1—Pd1—C18	86.37 (4)
O1—Pd1—O2	60.19 (4)
O1—Pd1—C18	159.59 (5)
O2—Pd1—C18	99.69 (5)

**Table 2:** Hydrogen-bond geometry (Å, °)

*D*—H⋯*A*	*D*—H	H⋯*A*	*D*⋯*A*	*D*—H⋯*A*
C4—H4*B*⋯O1	0.98	2.25	3.169 (2)	156

**Fig. 1.**
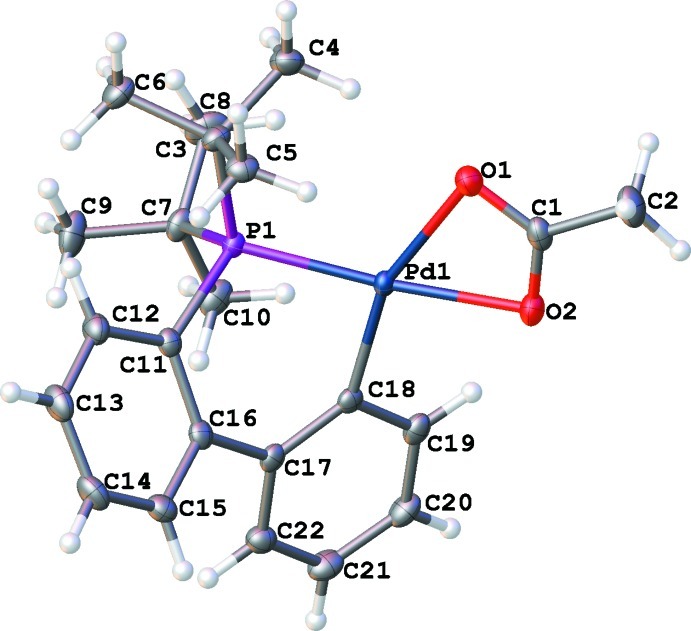
Molecular structure of (I) with anisotropic displacement ellipsoids drawn at the 50% probability level.

**Fig. 2.**
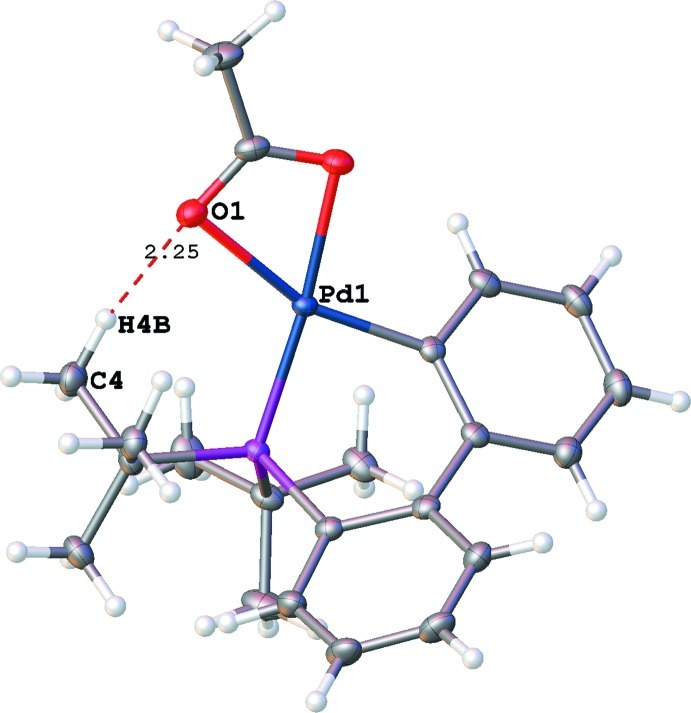
Diagram of (I) showing the weak C—H···O intramolecular interaction indicating the H···A distance of 2.25 Å.

## supplementary crystallographic information

### Comment

Our continued studies of palladium catalysed reactions (Ormondi *et al.*, 2011; Williams *et al.*, 2008; van Blerk & Holzapfel, 2009) includes a comparison of the efficiency of a wide range of palladocycles compared against the title compound (I), acetato-(2'-di-*t*-butylphosphanyl-1,1'-biphenyl-2yl)palladium(II). Compound (I) is an efficient and stable catalyst introduced by Zim & Buchwald (2005). We now report the crystal structure of (I) as part of a structure–activity study.

There is significant deviation from the ideal square planar geometry around the Pd atom as a result of its coordination to the acetate moiety. This distorted geometry is evident from the deviations in bond angles from 90°. The data in Table 1 demonstrate the deviations in the coordination geometry. A weak intramolecular C—H···O interaction is noted (Fig. 2 and Table 2).

### Experimental

A solution of 2-(biphenyl)-di-*tert*-butylphosphine (298 mg; 1 mmol) and palladium acetate (224 mg; 1 mmol) in 15 ml of chloroform was refluxed under argon for 3 h. The solvent was evaporated *in vacuo* to leave a colourless crystalline residue (464 mg; *ca* 100%). The solid was taken up in dichloromethane (3 ml) and the solution diluted with cyclohexane (10 ml). The solvent was allowed to slowly evaporate in a stream of nitrogen until the solution was reduced to 3 ml. This resulted in the formation of well formed yellow blocks of the title compound (I) (288 mg; m.p. 409–414 K).

### Refinement

The H atoms were included at idealized positions and were allowed to ride with C—H = 0.95–0.98 Å, and with *U*_iso_(H) = 1.2–1.5*U*_eq_(C). The structure was refined as a racemic twin precluding the determination of the absolute structure. Owing to poor agreement, the 0 1 6 and 1 4 0 reflections were omitted from the final refinement.

### Crystal data

**Table:**

[Pd(C_2_H_3_O_2_)(C_20_H_26_P)]	*F*(000) = 952
*M**_r_* = 462.82	*D*_x_ = 1.491 Mg m^−^^3^
Orthorhombic, *P*2_1_2_1_2_1_	Mo *K*α radiation, λ = 0.71073 Å
Hall symbol: P 2ac 2ab	Cell parameters from 9242 reflections
*a* = 9.800 (3) Å	θ = 2.5–28.4°
*b* = 14.2392 (4) Å	µ = 0.99 mm^−^^1^
*c* = 14.7772 (5) Å	*T* = 100 K
*V* = 2062.1 (6) Å^3^	Block, yellow
*Z* = 4	0.25 × 0.19 × 0.14 mm

### Data collection

**Table:**

Bruker APEXII CCD diffractometer	5179 independent reflections
Radiation source: fine-focus sealed tube	5077 reflections with *I* > 2σ(*I*)
Graphite monochromator	*R*_int_ = 0.018
φ andω scans	θ_max_ = 28.4°, θ_min_ = 2.0°
Absorption correction: multi-scan (AXSCALE; Bruker, 2010)	*h* = −13→12
*T*_min_ = 0.790, *T*_max_ = 0.874	*k* = −19→17
24048 measured reflections	*l* = −19→18

### Refinement

**Table:**

Refinement on *F*^2^	Secondary atom site location: difference Fourier map
Least-squares matrix: full	Hydrogen site location: inferred from neighbouring sites
*R*[*F*^2^ > 2σ(*F*^2^)] = 0.014	H-atom parameters constrained
*wR*(*F*^2^) = 0.034	*w* = 1/[σ^2^(*F*_o_^2^) + (0.0177*P*)^2^ + 0.2811*P*] where *P* = (*F*_o_^2^ + 2*F*_c_^2^)/3
*S* = 1.06	(Δ/σ)_max_ = 0.002
5179 reflections	Δρ_max_ = 0.27 e Å^−^^3^
243 parameters	Δρ_min_ = −0.19 e Å^−^^3^
0 restraints	Absolute structure: nd
Primary atom site location: structure-invariant direct methods	

### Special details

**Table:**

Geometry. All e.s.d.'s (except the e.s.d. in the dihedral angle between two l.s. planes) are estimated using the full covariance matrix. The cell e.s.d.'s are taken into account individually in the estimation of e.s.d.'s in distances, angles and torsion angles; correlations between e.s.d.'s in cell parameters are only used when they are defined by crystal symmetry. An approximate (isotropic) treatment of cell e.s.d.'s is used for estimating e.s.d.'s involving l.s. planes.
Refinement. Refinement of *F*^2^ against ALL reflections. The weighted *R*-factor *wR* and goodness of fit *S* are based on *F*^2^, conventional *R*-factors *R* are based on *F*, with *F* set to zero for negative *F*^2^. The threshold expression of *F*^2^ > σ(*F*^2^) is used only for calculating *R*-factors(gt) *etc*. and is not relevant to the choice of reflections for refinement. *R*-factors based on *F*^2^ are statistically about twice as large as those based on *F*, and *R*-factors based on ALL data will be even larger.

### Fractional atomic coordinates and isotropic or equivalent isotropic displacement parameters (Å^2^)

**Table:**

	*x*	*y*	*z*	*U*_iso_*/*U*_eq_	
C1	0.01684 (16)	0.64306 (10)	0.01415 (9)	0.0200 (3)	
C2	−0.03332 (19)	0.70910 (12)	−0.05813 (11)	0.0296 (4)	
H2A	0.0353	0.7580	−0.0688	0.044*	
H2B	−0.1189	0.7383	−0.0384	0.044*	
H2C	−0.0490	0.6740	−0.1142	0.044*	
C3	−0.12994 (14)	0.36953 (10)	0.17962 (10)	0.0179 (3)	
C4	−0.12773 (16)	0.35830 (11)	0.07523 (10)	0.0234 (3)	
H4A	−0.0721	0.3036	0.0590	0.035*	
H4B	−0.0886	0.4148	0.0477	0.035*	
H4C	−0.2211	0.3493	0.0530	0.035*	
C5	−0.22808 (14)	0.45126 (10)	0.20099 (10)	0.0207 (3)	
H5A	−0.1956	0.5085	0.1712	0.031*	
H5B	−0.2314	0.4613	0.2666	0.031*	
H5C	−0.3196	0.4358	0.1788	0.031*	
C6	−0.18496 (16)	0.27675 (11)	0.21887 (11)	0.0238 (3)	
H6A	−0.1824	0.2795	0.2851	0.036*	
H6B	−0.1283	0.2245	0.1978	0.036*	
H6C	−0.2792	0.2673	0.1987	0.036*	
C7	0.16700 (15)	0.30436 (10)	0.21904 (10)	0.0190 (3)	
C8	0.15467 (17)	0.24809 (11)	0.13080 (13)	0.0300 (4)	
H8A	0.1589	0.2911	0.0791	0.045*	
H8B	0.0675	0.2144	0.1301	0.045*	
H8C	0.2298	0.2029	0.1269	0.045*	
C9	0.13811 (18)	0.24130 (12)	0.30108 (13)	0.0299 (4)	
H9A	0.0436	0.2190	0.2985	0.045*	
H9B	0.1520	0.2772	0.3569	0.045*	
H9C	0.2003	0.1874	0.3002	0.045*	
C10	0.31527 (15)	0.33883 (11)	0.22510 (12)	0.0238 (3)	
H10A	0.3281	0.3739	0.2815	0.036*	
H10B	0.3352	0.3798	0.1735	0.036*	
H10C	0.3771	0.2848	0.2241	0.036*	
C11	0.03089 (14)	0.44692 (9)	0.33655 (8)	0.0154 (3)	
C12	−0.07717 (15)	0.41959 (11)	0.39277 (10)	0.0209 (3)	
H12	−0.1366	0.3708	0.3735	0.025*	
C13	−0.09954 (16)	0.46231 (12)	0.47641 (9)	0.0246 (3)	
H13	−0.1752	0.4443	0.5127	0.029*	
C14	−0.01070 (15)	0.53113 (13)	0.50607 (9)	0.0243 (3)	
H14	−0.0268	0.5620	0.5620	0.029*	
C15	0.10184 (17)	0.55503 (10)	0.45413 (9)	0.0201 (3)	
H15	0.1646	0.6003	0.4765	0.024*	
C16	0.12513 (13)	0.51377 (9)	0.36921 (9)	0.0163 (3)	
C17	0.24987 (13)	0.54179 (10)	0.31892 (9)	0.0157 (2)	
C18	0.25148 (14)	0.55169 (9)	0.22435 (9)	0.0147 (3)	
C19	0.37097 (15)	0.58258 (10)	0.18224 (10)	0.0190 (3)	
H19	0.3711	0.5933	0.1188	0.023*	
C20	0.48925 (15)	0.59791 (11)	0.23147 (11)	0.0221 (3)	
H20	0.5700	0.6179	0.2016	0.026*	
C21	0.48961 (15)	0.58397 (11)	0.32458 (11)	0.0237 (3)	
H21	0.5710	0.5926	0.3585	0.028*	
C22	0.36993 (14)	0.55730 (9)	0.36744 (10)	0.0204 (3)	
H22	0.3696	0.5494	0.4313	0.024*	
O1	−0.05113 (11)	0.56989 (7)	0.03223 (7)	0.0211 (2)	
O2	0.12585 (11)	0.66066 (7)	0.05752 (7)	0.0209 (2)	
P1	0.04521 (4)	0.40789 (2)	0.21866 (2)	0.01354 (7)	
Pd1	0.097889 (10)	0.536270 (7)	0.139786 (6)	0.01311 (3)	

### Atomic displacement parameters (Å^2^)

**Table:**

	*U*^11^	*U*^22^	*U*^33^	*U*^12^	*U*^13^	*U*^23^
C1	0.0253 (8)	0.0188 (7)	0.0158 (6)	0.0050 (6)	0.0024 (6)	0.0028 (5)
C2	0.0350 (9)	0.0291 (9)	0.0248 (8)	0.0055 (7)	−0.0017 (7)	0.0105 (7)
C3	0.0173 (7)	0.0148 (7)	0.0215 (7)	−0.0024 (5)	−0.0021 (5)	−0.0004 (5)
C4	0.0266 (9)	0.0214 (7)	0.0223 (7)	−0.0019 (6)	−0.0036 (6)	−0.0044 (6)
C5	0.0159 (6)	0.0219 (8)	0.0245 (7)	0.0010 (6)	−0.0035 (5)	−0.0011 (6)
C6	0.0204 (7)	0.0191 (7)	0.0318 (8)	−0.0050 (6)	0.0008 (6)	0.0012 (6)
C7	0.0188 (7)	0.0129 (7)	0.0253 (7)	0.0026 (5)	0.0034 (6)	0.0028 (5)
C8	0.0268 (8)	0.0208 (8)	0.0424 (10)	0.0046 (6)	0.0002 (8)	−0.0107 (7)
C9	0.0258 (8)	0.0221 (8)	0.0419 (9)	0.0061 (6)	0.0066 (7)	0.0153 (7)
C10	0.0191 (7)	0.0154 (7)	0.0367 (9)	0.0035 (6)	0.0024 (6)	0.0026 (6)
C11	0.0154 (6)	0.0157 (7)	0.0151 (6)	0.0031 (5)	−0.0001 (5)	0.0028 (5)
C12	0.0190 (8)	0.0245 (7)	0.0191 (6)	−0.0029 (6)	0.0006 (5)	0.0041 (5)
C13	0.0202 (6)	0.0363 (8)	0.0173 (6)	0.0004 (9)	0.0039 (6)	0.0052 (6)
C14	0.0253 (7)	0.0332 (8)	0.0144 (6)	0.0058 (7)	0.0015 (5)	0.0004 (7)
C15	0.0206 (6)	0.0227 (7)	0.0171 (6)	0.0017 (6)	−0.0037 (6)	−0.0005 (5)
C16	0.0164 (6)	0.0163 (6)	0.0162 (6)	0.0032 (5)	−0.0019 (5)	0.0043 (5)
C17	0.0168 (6)	0.0104 (6)	0.0199 (6)	0.0012 (5)	0.0009 (5)	0.0015 (5)
C18	0.0165 (6)	0.0093 (6)	0.0183 (6)	0.0014 (5)	−0.0005 (5)	0.0000 (5)
C19	0.0211 (8)	0.0124 (6)	0.0235 (7)	0.0004 (5)	0.0041 (6)	0.0006 (5)
C20	0.0152 (7)	0.0178 (7)	0.0331 (8)	−0.0019 (6)	0.0036 (6)	0.0009 (6)
C21	0.0160 (7)	0.0213 (8)	0.0338 (8)	−0.0006 (6)	−0.0039 (6)	−0.0008 (6)
C22	0.0211 (7)	0.0181 (7)	0.0220 (6)	0.0005 (5)	−0.0030 (6)	−0.0008 (5)
O1	0.0249 (5)	0.0200 (5)	0.0185 (5)	0.0001 (4)	−0.0028 (4)	0.0025 (4)
O2	0.0241 (6)	0.0174 (5)	0.0211 (5)	−0.0008 (4)	0.0010 (4)	0.0054 (4)
P1	0.01417 (16)	0.01141 (16)	0.01505 (15)	0.00016 (13)	0.00127 (13)	0.00109 (12)
Pd1	0.01529 (5)	0.01095 (4)	0.01310 (4)	0.00049 (4)	0.00091 (4)	0.00145 (4)

### Geometric parameters (Å, º)

**Table:**

C1—O1	1.2651 (18)	C10—H10B	0.9800
C1—O2	1.2707 (19)	C10—H10C	0.9800
C1—C2	1.506 (2)	C11—C12	1.401 (2)
C2—H2A	0.9800	C11—C16	1.4114 (19)
C2—H2B	0.9800	C11—P1	1.8339 (14)
C2—H2C	0.9800	C12—C13	1.395 (2)
C3—C6	1.540 (2)	C12—H12	0.9500
C3—C5	1.542 (2)	C13—C14	1.382 (2)
C3—C4	1.551 (2)	C13—H13	0.9500
C3—P1	1.8915 (16)	C14—C15	1.386 (2)
C4—H4A	0.9800	C14—H14	0.9500
C4—H4B	0.9800	C15—C16	1.4043 (19)
C4—H4C	0.9800	C15—H15	0.9500
C5—H5A	0.9800	C16—C17	1.4852 (19)
C5—H5B	0.9800	C17—C22	1.3954 (19)
C5—H5C	0.9800	C17—C18	1.4046 (18)
C6—H6A	0.9800	C18—C19	1.397 (2)
C6—H6B	0.9800	C19—C20	1.386 (2)
C6—H6C	0.9800	C19—H19	0.9500
C7—C9	1.535 (2)	C20—C21	1.390 (2)
C7—C8	1.535 (2)	C20—H20	0.9500
C7—C10	1.536 (2)	C21—C22	1.386 (2)
C7—P1	1.8968 (15)	C21—H21	0.9500
C8—H8A	0.9800	C22—H22	0.9500
C8—H8B	0.9800	Pd1—P1	2.2285 (7)
C8—H8C	0.9800	Pd1—O1	2.2109 (12)
C9—H9A	0.9800	Pd1—O2	2.1657 (11)
C9—H9B	0.9800	Pd1—C1	2.5278 (15)
C9—H9C	0.9800	Pd1—C18	1.9686 (15)
C10—H10A	0.9800		
			
O1—C1—O2	119.91 (13)	C7—C10—H10C	109.5
O1—C1—C2	119.48 (14)	H10A—C10—H10C	109.5
O2—C1—C2	120.60 (14)	H10B—C10—H10C	109.5
O1—C1—Pd1	60.99 (8)	C12—C11—C16	118.63 (13)
O2—C1—Pd1	58.94 (7)	C12—C11—P1	122.48 (11)
C2—C1—Pd1	177.93 (11)	C16—C11—P1	118.63 (10)
C1—C2—H2A	109.5	C13—C12—C11	121.54 (14)
C1—C2—H2B	109.5	C13—C12—H12	119.2
H2A—C2—H2B	109.5	C11—C12—H12	119.2
C1—C2—H2C	109.5	C14—C13—C12	119.42 (14)
H2A—C2—H2C	109.5	C14—C13—H13	120.3
H2B—C2—H2C	109.5	C12—C13—H13	120.3
C6—C3—C5	110.60 (12)	C13—C14—C15	119.98 (14)
C6—C3—C4	106.92 (12)	C13—C14—H14	120.0
C5—C3—C4	106.87 (12)	C15—C14—H14	120.0
C6—C3—P1	116.78 (10)	C14—C15—C16	121.43 (14)
C5—C3—P1	106.59 (10)	C14—C15—H15	119.3
C4—C3—P1	108.69 (10)	C16—C15—H15	119.3
C3—C4—H4A	109.5	C15—C16—C11	118.78 (12)
C3—C4—H4B	109.5	C15—C16—C17	117.94 (12)
H4A—C4—H4B	109.5	C11—C16—C17	123.28 (12)
C3—C4—H4C	109.5	C22—C17—C18	119.07 (13)
H4A—C4—H4C	109.5	C22—C17—C16	118.65 (12)
H4B—C4—H4C	109.5	C18—C17—C16	122.27 (12)
C3—C5—H5A	109.5	C19—C18—C17	118.95 (13)
C3—C5—H5B	109.5	C19—C18—Pd1	113.16 (10)
H5A—C5—H5B	109.5	C17—C18—Pd1	127.72 (10)
C3—C5—H5C	109.5	C20—C19—C18	121.12 (14)
H5A—C5—H5C	109.5	C20—C19—H19	119.4
H5B—C5—H5C	109.5	C18—C19—H19	119.4
C3—C6—H6A	109.5	C19—C20—C21	119.95 (14)
C3—C6—H6B	109.5	C19—C20—H20	120.0
H6A—C6—H6B	109.5	C21—C20—H20	120.0
C3—C6—H6C	109.5	C22—C21—C20	119.28 (14)
H6A—C6—H6C	109.5	C22—C21—H21	120.4
H6B—C6—H6C	109.5	C20—C21—H21	120.4
C9—C7—C8	110.54 (13)	C21—C22—C17	121.48 (14)
C9—C7—C10	108.38 (13)	C21—C22—H22	119.3
C8—C7—C10	106.90 (13)	C17—C22—H22	119.3
C9—C7—P1	109.94 (10)	C1—O1—Pd1	88.98 (9)
C8—C7—P1	110.71 (11)	C1—O2—Pd1	90.88 (9)
C10—C7—P1	110.30 (10)	C11—P1—C3	107.92 (7)
C7—C8—H8A	109.5	C11—P1—C7	106.31 (6)
C7—C8—H8B	109.5	C3—P1—C7	110.33 (7)
H8A—C8—H8B	109.5	C11—P1—Pd1	105.42 (5)
C7—C8—H8C	109.5	C3—P1—Pd1	106.72 (5)
H8A—C8—H8C	109.5	C7—P1—Pd1	119.56 (5)
H8B—C8—H8C	109.5	P1—Pd1—O1	113.61 (3)
C7—C9—H9A	109.5	P1—Pd1—O2	173.58 (3)
C7—C9—H9B	109.5	P1—Pd1—C18	86.37 (4)
H9A—C9—H9B	109.5	O1—Pd1—O2	60.19 (4)
C7—C9—H9C	109.5	O1—Pd1—C18	159.59 (5)
H9A—C9—H9C	109.5	O2—Pd1—C18	99.69 (5)
H9B—C9—H9C	109.5	C18—Pd1—C1	129.74 (5)
C7—C10—H10A	109.5	O2—Pd1—C1	30.17 (5)
C7—C10—H10B	109.5	O1—Pd1—C1	30.03 (4)
H10A—C10—H10B	109.5	P1—Pd1—C1	143.59 (4)
			
C16—C11—C12—C13	5.3 (2)	C4—C3—P1—C7	−77.17 (11)
P1—C11—C12—C13	−168.67 (11)	C6—C3—P1—Pd1	175.16 (10)
C11—C12—C13—C14	−2.1 (2)	C5—C3—P1—Pd1	−60.68 (10)
C12—C13—C14—C15	−2.0 (2)	C4—C3—P1—Pd1	54.18 (10)
C13—C14—C15—C16	2.9 (2)	C9—C7—P1—C11	40.82 (13)
C14—C15—C16—C11	0.3 (2)	C8—C7—P1—C11	163.24 (11)
C14—C15—C16—C17	−178.63 (13)	C10—C7—P1—C11	−78.65 (11)
C12—C11—C16—C15	−4.31 (19)	C9—C7—P1—C3	−75.95 (12)
P1—C11—C16—C15	169.90 (10)	C8—C7—P1—C3	46.47 (12)
C12—C11—C16—C17	174.56 (12)	C10—C7—P1—C3	164.59 (10)
P1—C11—C16—C17	−11.24 (18)	C9—C7—P1—Pd1	159.80 (9)
C15—C16—C17—C22	37.76 (18)	C8—C7—P1—Pd1	−77.78 (11)
C11—C16—C17—C22	−141.12 (14)	C10—C7—P1—Pd1	40.34 (12)
C15—C16—C17—C18	−143.27 (14)	C19—C18—Pd1—O2	−41.41 (11)
C11—C16—C17—C18	37.9 (2)	C17—C18—Pd1—O2	133.73 (12)
C22—C17—C18—C19	−4.0 (2)	C19—C18—Pd1—O1	−50.65 (19)
C16—C17—C18—C19	177.00 (12)	C17—C18—Pd1—O1	124.49 (14)
C22—C17—C18—Pd1	−178.92 (10)	C19—C18—Pd1—P1	140.72 (10)
C16—C17—C18—Pd1	2.1 (2)	C17—C18—Pd1—P1	−44.15 (12)
C17—C18—C19—C20	4.0 (2)	C19—C18—Pd1—C1	−44.46 (13)
Pd1—C18—C19—C20	179.56 (11)	C17—C18—Pd1—C1	130.67 (12)
C18—C19—C20—C21	−1.0 (2)	C1—O2—Pd1—C18	−175.32 (9)
C19—C20—C21—C22	−1.8 (2)	C1—O2—Pd1—O1	0.98 (8)
C20—C21—C22—C17	1.7 (2)	C1—O1—Pd1—C18	9.53 (17)
C18—C17—C22—C21	1.3 (2)	C1—O1—Pd1—O2	−0.98 (8)
C16—C17—C22—C21	−179.71 (13)	C1—O1—Pd1—P1	177.14 (7)
O2—C1—O1—Pd1	1.67 (13)	C11—P1—Pd1—C18	54.30 (6)
C2—C1—O1—Pd1	−177.69 (13)	C3—P1—Pd1—C18	168.90 (6)
O1—C1—O2—Pd1	−1.71 (13)	C7—P1—Pd1—C18	−65.13 (7)
C2—C1—O2—Pd1	177.65 (13)	C11—P1—Pd1—O1	−121.40 (6)
C12—C11—P1—C3	20.76 (14)	C3—P1—Pd1—O1	−6.80 (6)
C16—C11—P1—C3	−153.20 (11)	C7—P1—Pd1—O1	119.17 (7)
C12—C11—P1—C7	−97.61 (13)	C11—P1—Pd1—C1	−118.98 (7)
C16—C11—P1—C7	88.43 (12)	C3—P1—Pd1—C1	−4.39 (8)
C12—C11—P1—Pd1	134.52 (11)	C7—P1—Pd1—C1	121.59 (8)
C16—C11—P1—Pd1	−39.45 (11)	O1—C1—Pd1—C18	−175.69 (8)
C6—C3—P1—C11	−71.95 (12)	O2—C1—Pd1—C18	6.00 (11)
C5—C3—P1—C11	52.21 (11)	O1—C1—Pd1—O2	178.31 (13)
C4—C3—P1—C11	167.07 (10)	O2—C1—Pd1—O1	−178.31 (13)
C6—C3—P1—C7	43.81 (13)	O1—C1—Pd1—P1	−4.42 (11)
C5—C3—P1—C7	167.97 (9)	O2—C1—Pd1—P1	177.27 (6)

### Hydrogen-bond geometry (Å, º)

**Table:**

*D*—H···*A*	*D*—H	H···*A*	*D*···*A*	*D*—H···*A*
C4—H4*B*···O1	0.98	2.25	3.169 (2)	156
